# Predictive value of pedicle involvement with MRI in spine metastases

**DOI:** 10.18632/oncotarget.10884

**Published:** 2016-07-28

**Authors:** Wang Mi Liu, Rong Xing, Chong Bian, Yun Liang, Libo Jiang, Chen Qian, Jian Dong

**Affiliations:** ^1^ Department of Orthopedic Surgery, Zhongshan Hospital, Fudan University, Shanghai, China

**Keywords:** MRI, spine metastases, pedicle

## Abstract

**Objectives:**

The study aimed to retrospectively evaluate the accuracy and value of magnetic resonance imaging (MRI) in predicting pedicle involvement for patients with spine metastases.

**Methods:**

Forty-five patients with a vertebral metastasis encroaching at least one pedicle were studied using MRI before surgery and regularly after surgery. Patients were categorized on the basis of their numbers of pedicle involvement (Group 1: one pedicle was involved, n = 23; Group 2: two pedicles were involved, n = 22). The diagnostic accuracy was calculated, and comparisons of intraoperative blood loss and recurrence rate between the two groups were performed.

**Results:**

The overall performance of MRI in predicting the pedicle involvement was as follows: accuracy, 94.4%; sensitivity, 95.5%; and specificity, 91.3%. Less intraoperative blood loss was observed for Group 1 compared with Group 2 (1,661 ± 672 ml and 2,173 ± 790 ml, respectively, P = 0.024). Tumor relapse occurred in 8.7% (2/23) of Group 1 and in 22.7% (5/22) of Group 2 with median recurrence free survival time 14 and 9 months, respectively.

**Conclusions:**

MRI is a reliable approach to assess pedicle involvement. It has potential for use in the evaluation of the clinical characteristics of patients with spine metastases.

## INTRODUCTION

Bone is the third most common site of metastases in malignant tumors, and the spine is the most common site among bone metastases [1]. Because patients with spine metastases have a poor prognosis, these patients are a special group for spine surgeons, as well as other care providers. Given those patients who might be at high risk for receiving surgery, chemotherapy and stereotactic radiation therapy, past and present, are prevalent in clinical treatment guidance. However, with improvements in multidisciplinary care and surgical techniques, spine surgeons can treat spine metastases more effectively than before. Therefore, it is clear that a major change in the management of spine metastases is under way.

Among several surgical techniques, total en bloc spondylectomy (TES) of a metastatic spine tumor provides satisfactory neurologic outcomes and receivable local tumor control [2, 3]. However, the complex anatomy of the vertebrae, including structures such as vertebral body, pedicle, and vertebral arch, makes excision without transgression of the tumor difficult. It is especially true when a pedicle is encroached by tumor. For one vertebra, when only one pedicle is involved, spine surgeons are still able to perform TES with an oncological wide margin, whereas intralesional resection is inevitable when both pedicles are involved. Because post-operation rehabilitation of these patients is important for the quality of life during their remaining time, being aware of the precise position of tumor during preoperative evaluation enables the spine surgeons to make full preparations for the following surgery.

In view of high soft tissue contrast provided by MRI, MRI is optimal for lesions arising from the musculoskeletal system. Although MRI demonstrates limited anatomic detail about the cancellous bone of the vertebral body, it is sensitive to signal intensity change within the vertebral body, which represents pathologic tissue. Furthermore, during MRI examination, digital data on pedicles are obtained and translated into images. Therefore, MRI enables spine surgeons to be aware of whether one or both pedicles are involved before commencing surgery. In the present study, to evaluate the performance of MRI in predicting pedicle involvement and its predictive value, we reviewed retrospectively the records (intraoperative blood loss and local recurrence) and images of 45 patients in whom a metastatic spine tumor in one vertebral body was treated.

## RESULTS

**Figure 1:**
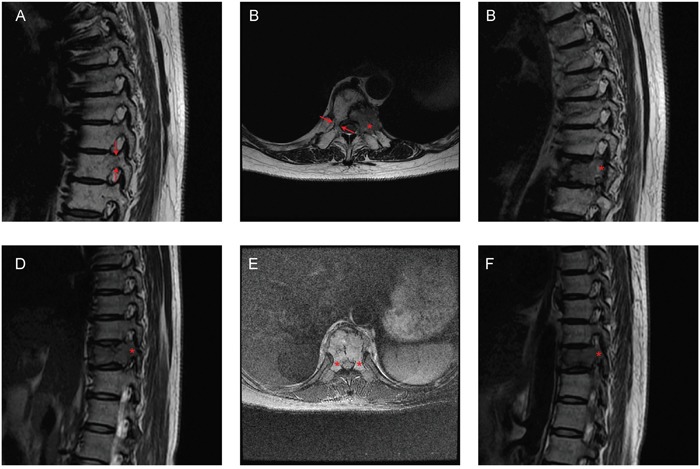
Representative images of Groups 1 and 2 **A-C**. A 60-year-old woman with T9 metastases from lung cancer. Only the left pedicle was involved in the sagittal (A, C) and transversal (B) MRI images. The patient was in Group 1. **D-F.** A 44-year-old woman with T10 metastases from breast cancer. Both pedicles were involved in the sagittal (D, F) and transversal **E.** MRI images. The patient was in Group 2. Arrows: pedicles with normal signal in MRI; Asterisks: pedicles with abnormal signal in MRI.

**Figure 2:**
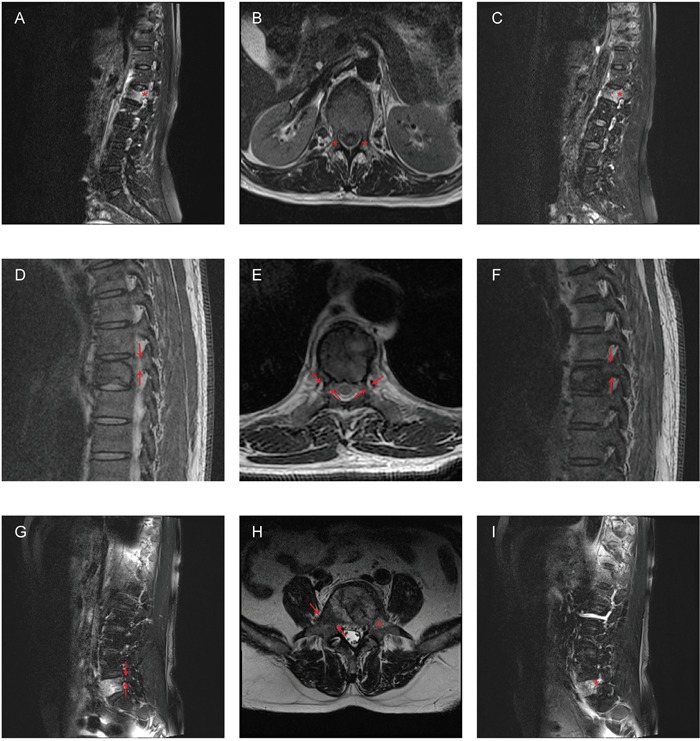
Examples of misdiagnosis according to MRI images **A-C**. A 58-year-old woman with L1 metastases from breast cancer. The patient only had the left pedicle involved, whereas both pedicles appeared involved in the sagittal (A, C) and transversal (B) MRI images. **D-F.** A 54-year-old man with L1 metastases from renal cell carcinoma. The patient had the left pedicle involved, whereas both pedicles appeared spared in the sagittal (D, F) and transversal (E) MRI images. **G-I**. A 67-year-old man with L5 metastases from lung cancer. The patient had both pedicles involved, whereas only the left pedicle appeared involved in the sagittal (G, I) and transversal (H) MRI images. Arrows: pedicles with normal signal in MRI; Asterisks: pedicles with abnormal signal in MRI.

Representative images of both groups are displayed in Figure 1. In Group 1, there were 23 involved and 23 uninvolved pedicles in 23 patients. Two patients were diagnosed as both pedicles being involved (Figure 2a-2c), and one patient was diagnosed as no pedicle being involved (Figure 2d-2f). The diagnostic performance of MRI in Group 1 was: accuracy, 91.6% (43/46); sensitivity, 95.7% (22/23); and specificity, 91.3% (21/23). In Group 2, there were 44 involved pedicles in 22 patients. Two patients were diagnosed as only one pedicle being involved (Figure 2g-2i). The diagnostic performance of MRI in Group 2 was: accuracy, 91.6% (42/44); and sensitivity, 91.6% (42/44). The overall performance of MRI in predicting the pedicle involvement was as follows: accuracy, 94.4%; sensitivity, 95.5%; and specificity, 91.3% (Table 1). We did not observe significant differences between MRI and pathology for detecting involved pedicles by the McNemar test (P = 1.000, Table 2). There was almost perfect agreement for the detection of the involvement of pedicles by MRI and pathology (Kappa = 0.86, P < 0.001; Table 2).

**Table 1:** Comparison of the involved pedicles findings*

Diagnostic characteristic	All
Accuracy	94.4%(85/90)
Sensitivity	95.5%(64/67)
Specificity	91.3%(21/23)

* Pathology was the standard of reference.

**Table 2:** Correlation between MRI diagnoses and pathological diagnoses

Open surgery	MRI	
	Positive	Negative
Positive	64	3
Negative	2	21

a McNemar test: P=1.000

b Kappa=0.86, P<0.001

**Figure 3:**
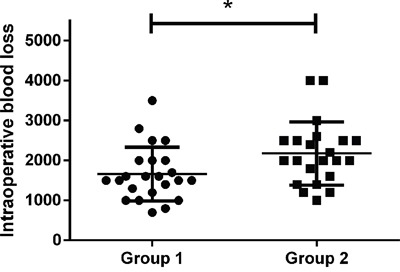
Intraoperative blood loss of the two groups The difference of intraoperative blood loss between the two groups was significant (*P = 0.024).

The average blood loss for all patients in Group 1 was 1,661 ± 672 ml, whereas it was 2,173 ± 790 ml for all patients in Group 2 (Figure 3). Therefore, compared with Group 2, there was significantly less intraoperative blood loss in Group 1 (P = 0.024).

**Figure 4:**
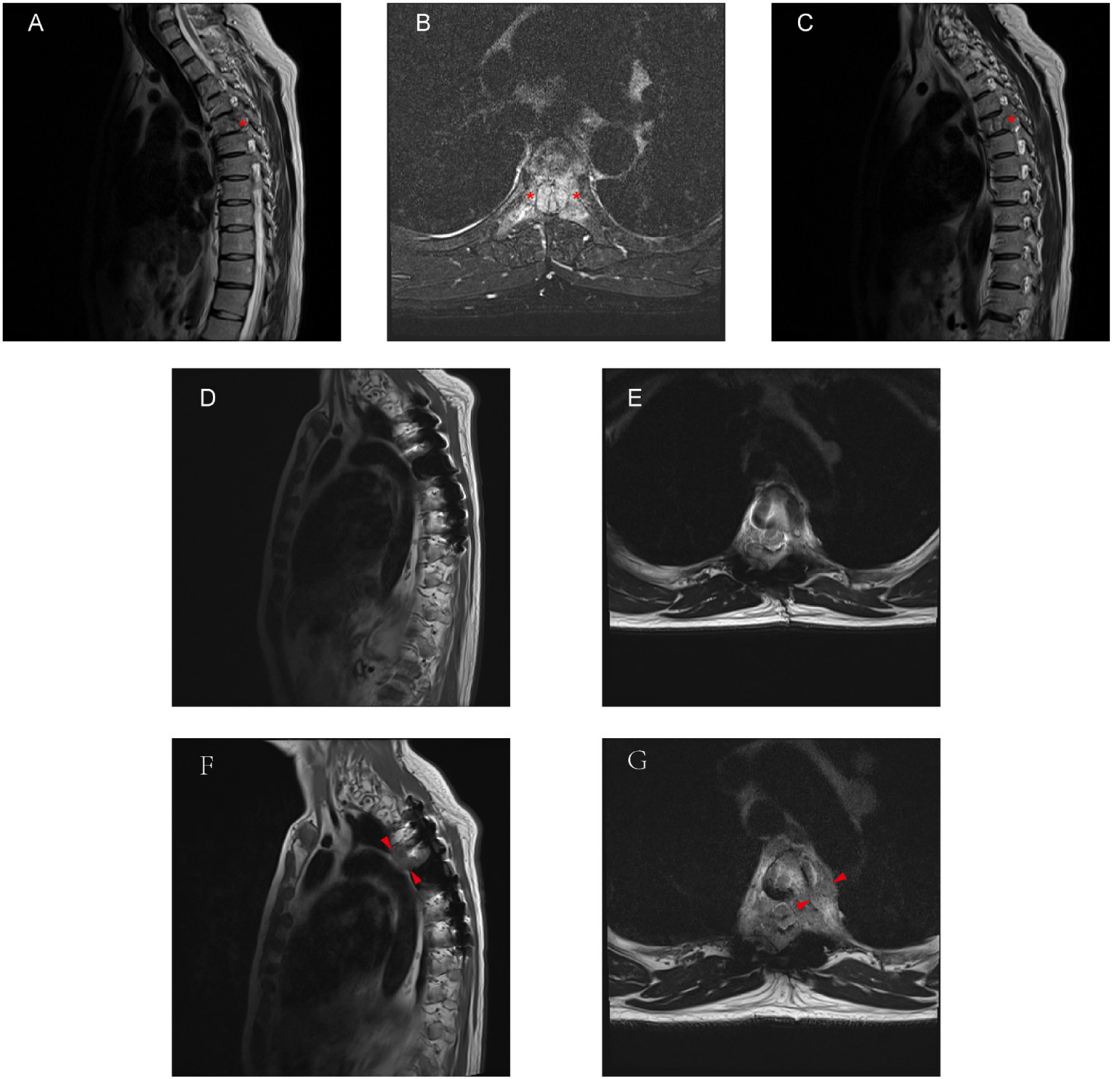
A 47-year-old man with T5 metastases from parotid acinic cell carcinoma **A-C**. Preoperative MRI displayed the lesion in the T5 vertebra, and both pedicles were involved in the sagittal (A, C) and transversal (B) MRI images. **D-E**. Six months after operation, MRI images showed no clue of the local recurrence. **F-G**. Thirty-six months later, as showed in the sagittal (F) and transversal (G) MRI images, there was a clear lesion site near the primary surgical region. Asterisks: pedicles with abnormal signal in MRI; Arrowheads: lesion site.

**Figure 5:**
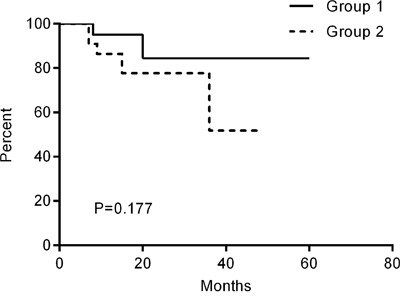
Kaplan-Meier curves of the recurrence free survival time

Local recurrence occurred in both groups. Figure 4 showed one case with local recurrence. Two (8.7%) of the 23 patients in Group 1 developed a local recurrence. The median recurrence free survival time for these two patients was 14 months (range 8–20 months). In Group 2, five (22.7%) of the 22 patients developed a local recurrence. The median recurrence free survival time for these five patients was 9 months (range 7–36 months). The difference between the curves of the two groups did not reach the significance level (Figure 5, P = 0.177).

## DISCUSSION

Data from autopsies on patients with cancer suggest the incidence of spinal metastases varies between 30% and 90% [4, 5]. Although there is a huge gap between these results, the risk of spine metastases has been increasing continuously as the life expectancy of patients with cancers is prolonged with the development of precise medicine, such as targeted therapy. However, all these patients are at risk for intractable pain, incontinence, and loss of mobility due to subsequent symptomatic spinal cord compression. For a long time, radiotherapy was proposed as the standard treatment for spine metastases by some authors [6, 7]. Therefore, surgery was the second-line management because adverse factors were described in these studies. However, in recent years, with the development of surgical techniques, TES has led to a better quality of life as a whole after surgery, compared to the previously used laminectomy [8]. As a consequence, TES has been increasingly adopted for the treatment of spine metastases among spine surgeons. The conclusion drawn from Patchell's study, that surgical treatment results in significantly increased survival time [9], is in contradiction with another report of no statistically significant difference between surgery and non-surgery in survival [10]. However, they both reported that surgical treatment can improve the quality of remaining life for patients with spine metastases. Of note, metastatic disease is generally incurable, especially spine metastases, so surgery mainly aims at prolonging survival and the palliation of symptoms. Because most patients with spine metastases are diagnosed at a late, but not terminal, stage, the risk of an extensive operative procedure, such as TES, must be weighed against only a small chance of improving life quality. Therefore, pre-operative prediction and planning play a vital role in obtaining an acceptable outcome for patients undergoing TES. As for TES, being aware of the involvement of pedicles before surgery is of importance for spine surgeons.

Since its application, MRI has provided the best evaluation of soft tissue pathology due to its excellent tissue contrast. Furthermore, images obtained from MRI sequences can be interpreted with radiographic and computerized tomography (CT) exams. Accordingly, spine surgeons are able to discover subtle osseous injury, such as edema, and determine the precise location of structural bony damage caused by tumor. As such, MRI is an ideal initial screening modality for patients with suspected spine metastasis. New and advanced MRI systems with higher field strengths and new pulse sequences have been applied to improve diagnostic performance in patients with spine metastasis. One of the important pulse sequences in musculoskeletal tissue is the short-tau inversion-recovery sequence with fat suppression, which could provide additional information about areas of fractures, bone bruising, and tumors neglected in radiography; it is ideal as a useful tool to inspect marrow in vertebrae [11]. Nonetheless, some patients with spinal metastasis have a sudden onset of fracture and rapid development of neurologic deterioration and may need surgery immediately. Therefore, they do not have time to wait for MRI systems with higher field strengths or receive extra pulse sequence examination. It is more rational for spine surgeons to assign standard MRI exams of the spine, which typically include T1- and T2-weighted images. Most, but not all, spine metastases on MRI is hypointensity on T1-weighted images, hyperintensity on T2-weighted images, and enhancement after contrast injection [12]. Because both osteoporosis and spinal metastases show hypointensity on T1-weighted images [13], the T2-weighted technique is the preferred sequence for confirming the involvement of pedicles. As for contrast injection, due to the above-mentioned emergency, some patients are not referred to accept this procedure. Therefore, we chose T2-weighted images and studied its performance in judging the involvement of pedicles.

In this study, the accuracy, sensitivity, and specificity of MRI to detect pedicle involvement were all favorable. We believe that the satisfactory diagnostic performance may be attributed to the following factors. First, MRI offers superior bone marrow and soft-tissue contrast to radiographic and CT exams. Thus, MRI has a significantly higher sensitivity than fluoroscopy and CT in displaying spine bone marrow lesions. In some cases, such lesions without obvious bone destruction, which would be omitted by radiographic and CT exams, are more likely to be detected by MRI [14]. Second, given the multi-planar imaging capabilities of MRI, the accurate lesion localization is beneficial to decide whether pedicles are involved. Furthermore, MRI does not require patients to be exposed to ionizing radiation. Although the ability of positron emission tomography (PET) or bone scans to identify metastatic lesions in the whole body is acceptable, their performance is inferior to MRI in detecting spine metastasis [15]. Therefore, this modality of MRI examination appears promising. However, there were still false-negative and false-positive involved pedicles judged by MRI in our study. These false identifications could be attributed to some appearance of common image artifacts, such as chemical shift and partial volume [11]. It also raises the possibilities that the degree of signal change in pedicles due to lesions is not significant enough to be detected by the MRI system used in our study. Thus, optimized sequences and high-field MR scanners, which provide higher image resolution and contrast with functional imaging, could resolve these problems [14].

Because metastatic disease is systemic dissemination and generally incurable, surgeons tend to adopt treatments that would palliate symptoms and prolong survival, if possible simultaneously. In the past, the only surgical procedure performed for spine metastases was laminectomy. However, most spine metastases are located within the vertebral body [16], rendering laminectomy inadequate in this circumstance. On the contrary, laminectomy not only does not remove the tumor completely but also increases the risk of instability. Because of its limitations, controversy over its application has existed for decades [7, 17]. In 1994, Tomita et al. reported a new surgical technique—TES—which signifies a new era of surgery in spine metastases [3, 18]. One area that has received much attention recently is the use of TES in the resection of multiple spine metastases with good prognostic characteristics [19]. However, surgery must not accelerate the deterioration of the remaining quality of life. It has been suggested that TES should be reserved for patients with life expectancy longer than 3 months and enough ability to tolerate the procedure [20, 21]. The patients in this study all satisfied the above indications. However, compared with a simpler palliative debulking procedure, the more aggressive TES is associated with increased complication rates, which can be as high as 33% [22]. Complications can quickly negate any intended benefit for these patients' remaining quality and even induce postoperative death. Thus, it is imperative for surgeons to be aware of the involvement of pedicles for every patient when TES will be scheduled later. This estimation is often made according to imaging examination. Determining the correct location of the lesion is important to avoid intralesional excision when only one pedicle is involved. Consequently, less intraoperative blood loss would be achieved through cutting off the pedicle that is not encroached by cancer, making the procedure straightforward and precise. Because a good general medical status benefits patients after surgery, less intraoperative blood loss would improve outcome to outweigh the efforts and costs of TES [23]. For those patients with both pedicles involved, spine surgeons should fully appreciate the potential risk of increased intraoperative blood loss. Full preoperative preparation refines manipulation during surgery, and tight tutelage in perioperative care is required.

There is a growing body of literature on the usefulness of MRI in assessing clinical progression from time of diagnosis to postoperative follow-up for patients with cancers [24], as well as spine metastases [25]. The most reliable signs of a malignant morphology of spine are paravertebral soft-tissue masses and infiltration of posterior elements [13]. Thus, every 6 months after the surgery (until death) or when suspicious symptoms occur, MRI of the patients in our study was performed. The local recurrence rate when only one pedicle was involved is relatively lower than that when both pedicles were involved, but this trend is not statistically significant. It may be just because of the small sample size in the cohort. Therefore, studies with large sample sizes will confirm this statistical significance. However, with the help of MRI, surgeons would become more aware of the potential risks and benefits of surgical options under different circumstances.

The limitations of our study are the retrospective nature of the research and low patient numbers with a variety of solid tumors. However, performing a randomized and prospective study in this field is difficult. There would be time bias because such a study needs a long time span to collect enough cases. A selection bias may have occurred in our study because we included only patients with one vertebral body lesion. In addition, as a confounder, the different vascularization degree among metastatic spine tumors due to the different types of primary cancers could impact the comparison of blood loss between the two groups. Therefore, because of the heterogeneity of the patient populations in the study, it is difficult to come to generalized conclusions. However, we advocate that MRI is a reliable instrument for assessing pedicle involvement, according to our study. In the future, a firm conclusion could be drawn by well-designed prospective and multi-center trials, including stratification of patients by histology as well.

In conclusion, these findings from our study confirm that MRI is an accurate and reliable diagnostic technique to evaluate the involvement of pedicles in spine metastases. It can be an invaluable tool to inform various clinical decision making in patients with spine metastases, such as planning surgery, discussing surgical outcomes, and making suggestions for future research.

## MATERIALS AND METHODS

### Patients

This study was approved by the Institutional Ethical Committee. Between January 2009 and December 2014, 45 patients with one vertebral metastasis but without metastasizing to other parts, who could tolerate the surgery and had a life expectancy of more than 3 months, were treated at the Department of Spine Oncology Surgery at our institution. All TES procedures were performed with a uniform technique by one team of surgeons in our institution. All lesions were histologically confirmed as metastatic spine tumors after surgical excision. Depending on their primary cancer and general health condition, all patients subsequently received different postoperative treatments, including chemotherapy, radiotherapy, hormone therapy, and bisphosphonate therapy or combined, when necessary. Of these 45 patients, 23 patients (Group 1) had one pedicle involved and 22 patients (Group 2) had both pedicles involved. There were 15 male and 8 female patients in Group 1, whereas there were 13 male and 9 female patients in Group 2. The median age at the time of the first diagnosis was 58 years (range, 24 to 76 years) for Group 1, 56.5 years (range, 20 to 73 years) for Group 2, and 57 years (range, 20 to 76 years) overall. In Group 1, eight patients had metastases from lung cancer, six from liver cancer, three from thyroid cancer, three from breast cancer, two from prostate cancer, and one from renal cell carcinoma. In Group 2, primary malignancies included breast cancer in eight patients, lung cancer in four, prostate cancer in four, liver cancer in three, renal cell carcinoma in two, and parotid acinic cell carcinoma in one (Table 3). The levels of the lesion ranged from T3 to L3 in Group 1 and from T4 to L5 in Group 2.

**Table 3:** Clinical profile of the patients in the two groups

Histopathological Diagnoses	No. of patients(%)
Group 1	23
Lung cancer	8 (35%)
Liver cancer	6 (26%)
Thyroid cancer	3 (13%)
Breast cancer	3 (13%)
Prostate cancer	2 (9%)
Renal cell carcinoma	1 (4%)
Group 2	22
Breast cancer	8 (36%)
Lung cancer	4 (18%)
Prostate cancer	4 (18%)
Liver cancer	3 (14%)
Renal cell carcinoma	2 (9%)
Parotid acinic cell carcinoma	1 (5%)

### MRI acquisition and analysis

All patients were placed supine, and the lesion area of the spine was imaged using a 1.5T scanner (Avanto Magnetom, Siemens Healthcare, Erlangen, Germany). Sagittal and transversal T2-weighted MRIs were selected for evaluation of the signal changes in pedicles. Two blinded, specially trained raters, who were not aware of the patients' clinical data and the involvement of the pedicles confirmed by pathology, independently performed the measurements on the relevant images to evaluate the involvement of the pedicles according to the signal changes in the pedicles. Pedicle fracture and tumor invasion into pedicle cortical bone were also classified as pedicle involvement. If there was discrepancy between the two raters, re-evaluation and joint decision ensued.

### Clinical evaluation

All patients received an MRI examination before surgery and every 6 months after surgery during follow-up. If recurrence was suspected when patients complained of back pain or radicular pain after surgery, MRI was prescribed immediately. Involvement of the pedicles was confirmed after surgery by pathological results. Intraoperative blood loss was recorded for every patient, whereas recurrence was identified when patients had a new neoplasm near the previous operation site confirmed by MRI.

### Statistical analysis

The MRI predictive accuracy, sensitivity, and specificity, in terms of the involvement of the pedicles, were calculated by comparing its diagnoses with diagnoses from pathology. The McNemar test was applied to determine the differences between the two methods. Cohen's weighted Kappa was used to evaluate the agreement between the diagnostic rates of MRI and pathology. The degree of agreement was interpreted as slight (k < 0.20), fair (k = 0.21−0.40), moderate (k = 0.41−0.60), substantial (k = 0.61−0.80), and almost perfect (k = 0.81−1.00). Intraoperative blood loss was represented as mean ± standard deviation for each group, and the statistical difference was evaluated with one-way analysis of variance (ANOVA). Recurrence free survival time was analyzed by the Kaplan-Meier method, and the log-rank test was adopted to compare different curves. P < 0.05 indicated the statistical significance. Data analyses were performed with SPSS 22.0 software.
